# Small Complex Rearrangement in *HINT1*-Related Axonal Neuropathy

**DOI:** 10.3390/genes15111483

**Published:** 2024-11-19

**Authors:** Alessandra Tessa, Mariapaola Schifino, Eliana Salvo, Rosanna Trovato, Luca Cesana, Silvia Frosini, Rosa Pasquariello, Giada Sgherri, Roberta Battini, Maria Clara Bonaglia, Filippo Maria Santorelli, Guja Astrea

**Affiliations:** 1 Molecular Medicine for Neurodegenerative and Neuromuscular Diseases Unit, IRCCS Stella Maris Foundation, 56128 Pisa, Italy; alessandra.tessa@fsm.unipi.it (A.T.); rosanna.trovato@fsm.unipi.it (R.T.); filippo.santorelli@fsm.unipi.it (F.M.S.); 2 Department of Neuroscience, IRCCS Stella Maris Foundation, 56128 Pisa, Italy; mariapaola.schifino@fsm.unipi.it (M.S.); silvia.frosini@fsm.unipi.it (S.F.); rosa.pasquariello@fsm.unipi.it (R.P.); giada.sgherri@fsm.unipi.it (G.S.); roberta.battini@fsm.unipi.it (R.B.); 3 Cytogenetics Laboratory, Scientific Institute, IRCCS Eugenio Medea, 23842 Bosisio Parini, Italy; eliana.salvo@lanostrafamiglia.it (E.S.); luca.cesana@lanostrafamiglia.it (L.C.); mariaclara.bonaglia@lanostrafamiglia.it (M.C.B.); 4 Department of Clinical and Experimental Medicine, University of Pisa, 56126 Pisa, Italy

**Keywords:** HINT1, axonal neuropathy, neuromyotonia, ARAN-NM

## Abstract

Background: Autosomal recessive inherited pathogenetic variants in the histidine triad nucleotide-binding protein 1 (*HINT1*) gene are responsible for an axonal Charcot-Marie-Tooth neuropathy associated with neuromyotonia, a phenomenon resulting from peripheral nerve hyperexcitability that causes a spontaneous muscle activity such as persistent muscle contraction, impaired relaxation and myokymias. Methods: Herein, we describe two brothers in whom biallelic *HINT1* variants were identified following a multidisciplinary approach. Results: The younger brother came to our attention for clinical evaluation of moderate intellectual disability, language developmental delay, and some behavioral issues. His elder brother presented mild intellectual disability, hyperactivity, tiptoe walking, and gait ataxia. At first evaluation, motor impairment with frequent falls, pes cavus, and distal hyposthenia with reduced osteotendinous reflexes were found in both. Grip myotonic phenomenon was also noted. Blood tests revealed mildly elevated creatine kinase, and neurophysiology investigations revealed predominantly axonal polyneuropathy. Muscle MRI highlighted fibro-adipose infiltration, prevalent in the lower limbs. Gene panel testing detected a heterozygous *HINT1* variant (c.355C>T/p.(Arg119Trp)) on the paternal allele. A further in-depth analysis using Integrative Genomics Viewer and Optical Genome Mapping led us to identify an additional variant in *HINT1* represented by a complex rearrangement located in the region 5′UTR-exon 1-intron 1, not previously described. Conclusions: This complex rearrangement could have been overlooked if the clinical picture had not been evaluated as a whole (from a clinical, neurophysiological, and neuroimaging point of view). Neuropsychiatric manifestations (intellectual disability, hyperactivity, etc.) are part of the picture of *HINT1*-related neuromyotonia.

## 1. Introduction

Hereditary peripheral neuropathies (HPN) are a group of multiple genetic conditions affecting predominantly the peripheral motor, sensory, and autonomic nerves, characterized by muscle weakness, worsening of motor abilities, and sensitive perception, with a degenerative trend of progression from the peripheral limbs upwards. The most common types of HPN are the Charcot-Marie-Tooth (CMT) disorders, a wide spectrum of neuropathic disorders, also known as primary hereditary motor sensory neuropathies, caused in 90% of patients by mutations in the PMP22, MFN2, MPZ, or GJB1 genes, usually autosomal dominant inherited; other genes are involved in the remaining 10% [1]

Recessive loss-of-function variants in the histidine triad nucleotide-binding protein 1 (*HINT1*) gene are associated with a common form of Charcot-Marie-Tooth (CMT), representing 2% of all CMT cases and about 10–12% of recessive CMTs [2], firstly described in 2012 [3]. It is an axonal, predominantly motor neuropathy associated in most patients with neuromyotonia, a hyperexcitability phenomenon of peripheral nerves consisting of spontaneous muscle activity such as persistent muscle contraction, impaired relaxation, and myokymias. This occurs more commonly in the hands; it is accentuated by the execution of action [4], and it is different from myotonia, which occurs after voluntary muscle contractions [5]. In some cases, the muscle activity is noticeable clinically, but sometimes it is only detectable by electromyographic (EMG) recording, which shows continuous and irregular doublet, triplet, or multiplet single motor unit discharges with high frequency (30–300 Hz) [3]. Symptoms usually start in the first decade of life, but a later disease onset has also been described [6], slowly progressing, with possible loss of ambulation during middle age. Brain MRI usually does not show abnormalities. Recently, neuropsychiatric disorders have been reported in patients with recessive *HINT1* variants, expanding the phenotypic spectrum of this condition; in particular, Morel et al. found an increased incidence of neurodevelopmental and psychiatric features such as intellectual deficiency (ID), attention deficit hyperactivity disorder (ADHD), generalized anxiety disorder (GAD), obsessive-compulsive disorder (OCD), and mood disorders [3]. Studies conducted on *HINT1* knocked-out (KO) mice showed a reduction in social behaviors and an increase in anxiety-related behaviors, aggressiveness, and schizophrenic traits [7], suggesting a significant role of this gene in the regulation of mood and behavior [8]. The precise recognition of the subtle neurological signs requires a careful clinical evaluation to establish the correct diagnostic pathway.

## 2. Materials and Methods

### 2.1. Participants and Clinical Assessment

Herein, we describe two brothers aged 11 (P1) and 16 years (P2), respectively, at the time of our evaluations. We acquired clinical data and used standardized scales to define the neurological, neuromotor, and neuropsychological characteristics of our patients. For neurological and neuromotor assessment, we used the Medical Research Council Scale (MRC-S) [9]. For neuropsychological evaluation, we used the Wechsler Intelligence Scale for Children (WISC-IV) [10] and psychological assessment questionnaires (CBCL 6–18 years [11] and MASC 2 [12]). Screening blood tests, including blood count with leukocyte formula, liver and kidney function, and creatine kinase dosage, were performed. Having obtained written informed consent, we collected blood DNA from patients and their parents.

### 2.2. Neurophysiology and Neuroimaging

A nerve conduction study (NCS) was conducted according to standard procedure. A 3-Tesla muscular magnetic resonance imaging (MRI) of the pelvis, right and left thigh, and right and left leg was performed using the following sequences: T1-weighted (T1W) axial fast spin echo (FSE), axial FSE-Short tau inversion recovery (STIR), and T1W axial FLEX sequences. Both the exams were performed at the age of 10 (P1) and 15 (P2) years old.

### 2.3. Next-Generation Sequencing (NGS)

DNA extraction was performed on peripheral blood samples obtained from the patients and their parents using MagCore Nucleic Acid Extractor (Diatech Lab Line, Jesi, Ancona, Italy). A targeted multigene resequencing panel (SureSelect; Agilent, Santa Clara, CA, USA) including 138 genes related to neuromuscular disorders (full list of genes available upon request) was performed in the index case (P1) using methodologies reported elsewhere [13]. Alignment of raw paired-end reads to the reference genome (version hg19) was performed with BWA Enrichment (v2.1.2). Variant calling was obtained using the Genome Analysis ToolKit (GATK). Sequencing data were filtered using the Expert Variant Interpreter-eVai v3.3 (evai.engenome.com; enGenome srl, Pavia, Italy). Variants were prioritized using the following parameters: non-synonymous exonic or ±5 bp intronic variants, minor allele frequency in the Genome Aggregation Database (GnomAD) of less than 0.01 (1%), quality of the call variant, coverage of ≥20×, mutation effect by CADD-Phred prediction tool (>23), Revel score >0.5. Synonymous variants and deep intronic variants were assumed to be benign or likely benign. The following public databases, accessed on 31 January 2024, were used for the interpretation of the variants: ClinVar (https://www.ncbi.nlm.nih.gov/clinvar/, accessed on 31 January 2024), LOVD (https://databases.lovd.nl/shared/genes, accessed on 31 January 2024), the Human Genome Mutation Database (HGMD, https://www.hgmd.cf.ac.uk/, accessed on 31 January 2024), Varsome (https://varsome.com/, accessed on 31 January 2024), Franklin by Genoox (https://franklin.genoox.com/clinical-db/home, accessed on 31 January 2024), and MobiDetails (https://mobidetails.iurc.montp.inserm.fr/MD/, accessed on 31 January 2024).

Finally, for the interpretation of the variants, the American College of Medical Genetics and Genomics (ACMG) 2015 guidelines were used [14]. Candidate variants were then inspected with the Integrative Genomics Viewer (IGV) and then confirmed by Sanger sequencing. To confirm the missense variant c.355C>T [p.(Arg119Trp)] in *HINT1,* we performed sequencing of exon 3, while for the rearrangement, we designed primers flanking the rearranged region reconstructed from mutated paired reads DNA obtained from the bam file.

### 2.4. Optical Genome Mapping (OGM) in the Family

OGM was performed according to the manufacturer’s instructions. In brief, ultra-high molecular weight (UHMW) gDNA (>150 kb) was extracted from the peripheral blood (EDTA) of the two brothers and their parents using SP Blood & Cell Culture DNA Isolation Kit (Bionano Genomics, San Diego, CA). gDNA was labeled according to the manufacturer’s instructions using the Bionano Prep Direct Label and Stain (DLS) Protocol and the data were analyzed on a Saphyr instrument (Bionano Genomics). A minimum of 500 Gb of data was acquired. Bionano Solve v3.8.2 was used to perform the de novo genome assembly at 80× coverage, trio-analysis, dual-analysis, variant calling, and annotation with default settings. Annotated variants were filtered for rare events (≤1% OGM control database), as described previously [15].

## 3. Results

### 3.1. Clinical Presentation

The two brothers, P1 and P2, were born from non-consanguineous parents. Family history is negative for neurological disorders. Antenatal information was significant for intrauterine growth retardation (IUGR) during both pregnancies. P2 also presented congenital non-functioning hypoplastic right kidney, observed during pregnancy ultrasound, along with placenta previa. Both brothers achieved regular motor development, but their parents noted mild incoordination during childhood. The index case (P1) presented a language developmental delay, which evolved into a clear, predominantly receptive language disorder. Both children exhibit ADHD symptoms and learning difficulties; neuropsychological evaluation later confirmed the presence of intellectual disability in both. P1 showed behavioral difficulties, such as aggressiveness and oppositional defiant behaviors. Because of behavioral and cognitive difficulties, he initially underwent an etiological assessment with creatine and guanidoacetic acid blood dosage, CGH-Array, and *FMR1* gene analysis, all of which gave normal results. A previous brain MRI did not show significant elements except for a specific venous anomaly. At the age of 10 years old, P1 experienced rapid fatigue, stiffness in the lower limbs, nocturnal cramps, and difficulty walking. Similar symptoms occurred in his brother during adolescence.

The clinical evaluation of P1 revealed stiffness of the tibiotarsal joint, slight accentuation of muscular trophism of suras, and mild weakness of the pelvic girdle (MRC4). During dorsiflexion of the foot, a similar level of weakness (MRC4) was observed bilaterally, along with a reduction in distal osteotendinous reflexes. Additionally, there was modest hyperlordosis and left scoliotic posture. The patients also exhibited difficulty in releasing the object. Fine motor tests indicated the presence of distal tremors.

The neurological examination in P2 revealed several findings, including distal hypotrophy in the lower limbs with increased consistency of the gastrocnemius muscles and stiffness, non-evocative distal reflexes, pelvic girdle weakness (MRC4), and significant difficulty in foot dorsiflexion (MRC1), resulting in foot drop, weakness of the thumb extensor (left > right) with retraction, pes cavus, myotonic-like phenomenon bilaterally in the hands, and distal tremors.

Neuropsychological assessment revealed moderate intellectual disability in P1 and mild impairment in P2. The cognitive profile and behavioral assessments of the two brothers are summarized in Table 1.

Blood tests revealed mild hyperCKemia (Proband 605 U/L, Patient2 1437 U/L). The complete blood count and metabolic panel were in the normal range. Analysis for CTG repeat expansion in the *DMPK* gene was in the normal range, too.

### 3.2. Neurophysiology and Neuroimaging

A picture compatible with predominantly axonal polyneuropathy was observed in nerve conduction study registration (NCS) in both subjects (Table 2).

**Figure 1:**
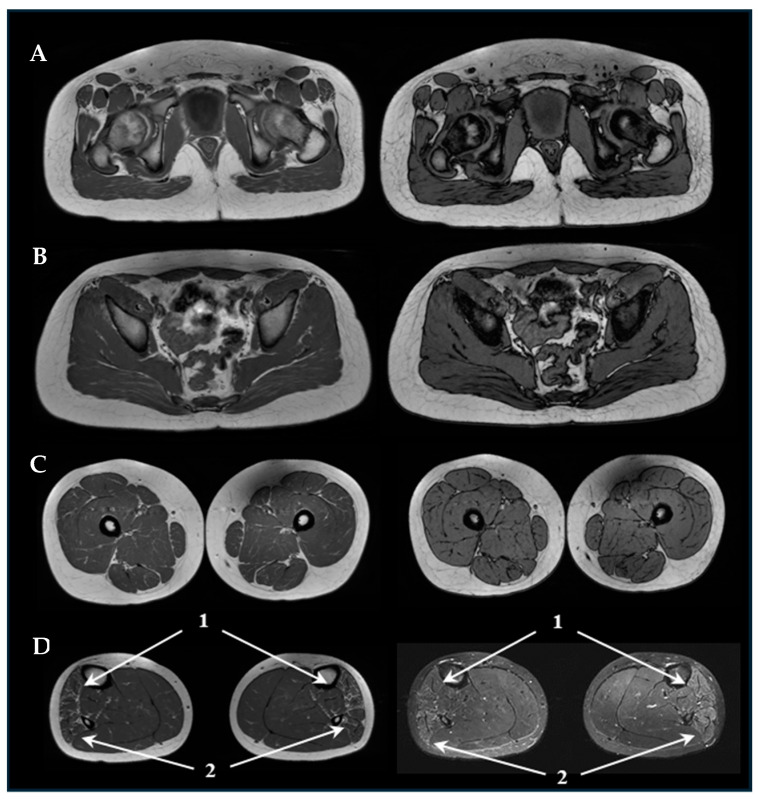
Muscle MRI of P1: on the left, Axial T1w sequences of pelvis (**A**), hip region (**B**), thighs. (**C**), and legs (**D**). On the right, T1 flex sequences of pelvis (**A**), hip region (**B**), and thighs (**C**) and FSE T1 STIR sequences of legs (**D**). The arrows highlight distal bilateral fibro-adipose infiltration of the anterior tibialis (1) and the peroneal group (2) in both sequences.

**Figure 2:**
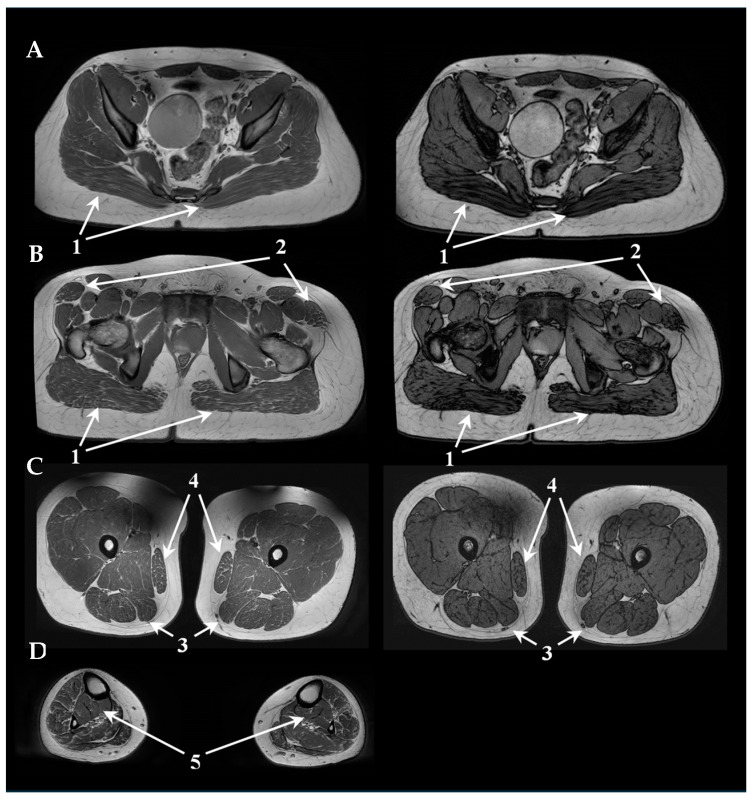
Muscle MRI of P2: on the left, Axial T1w sequences of pelvis (**A**), hip region (**B**), thighs (**C**), and legs (**D**). On the right, T1 flex sequences of pelvis (**A**), hip region (**B**), and thighs (**C**). The arrows highlight bilateral gluteus maximus (1) and tensor fasciae lata (2) infiltration, thigh posterior region (3) and gracilis (4) minimum substitution, reduction of leg trophism, diffuse muscular involvement with the exception of posterior tibialis and minimum soleus engagement (5).

A muscle MRI (pelvis, right and left thigh, and right and left leg) showed in P1 (Figure 1) distal bilateral, modest, and symmetrical fibro-adipose infiltration of the anterior tibialis and the peroneal group, reduction in muscle volume, and signs of muscle edema, whereas myoimaging in P2 (Figure 2) revealed mild bilateral fibro-adipose infiltration of gluteus maximus and tensor fasciae lata, minimum infiltration of thigh posterior region and gracilis, reduction in leg trophism with diffuse muscular involvement with the exception of posterior tibialis and minimum soleus engagement.

### 3.3. Molecular Genetics Investigations

**Figure 3:**
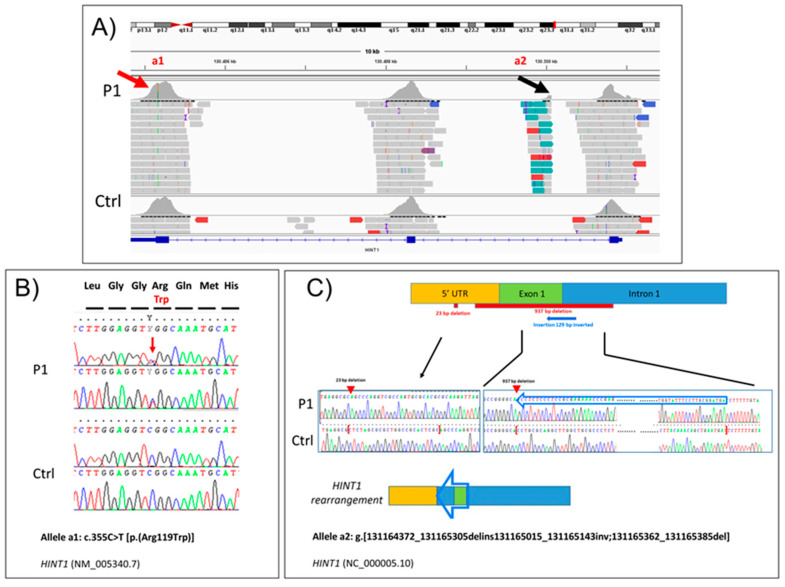
(**A**) Next-generation sequencing (NGS) visualization with the IGV of the heterozygous variant (a1) c.355C>T [p.(Arg119Trp)] in *HINT1* exon 3 (NM_005340.7) (red arrow) and of the rearrangement in region 5′UTR-exon1-intron1 (a2) (black arrow) visualized as a split and many read pairs with the same orientation suggesting the presence of a small inversion. (**B**) Electropherogram of Sanger sequencing confirms the c.355C>T [p.(Arg119Trp)] variant (a1) in P1. The arrow indicates the heterozygous variant compared to normal control (Ctrl). The corresponding codons are superimposed. (**C**) A schematic representation of the complex rearrangement. (**top**) Red boxes show deleted regions (23 bp in 5′UTR and 937 bp in 5′UTR-exon1-intron1), and the blue arrowed box shows the inverted region at the end of exon1 and initial part of intron 1. Electropherogram of Sanger sequencing (**middle**) of complex rearrangement (a2) located in the *HINT1* region 5′UTR-exon 1-intron 1 was detected as g.[131164372_131165305delins131165015_131165143inv;131165362_131165385del] (NC_000005.10). (**bottom**) Schematic proposed representation of the rearrangement.

**Figure 4:**
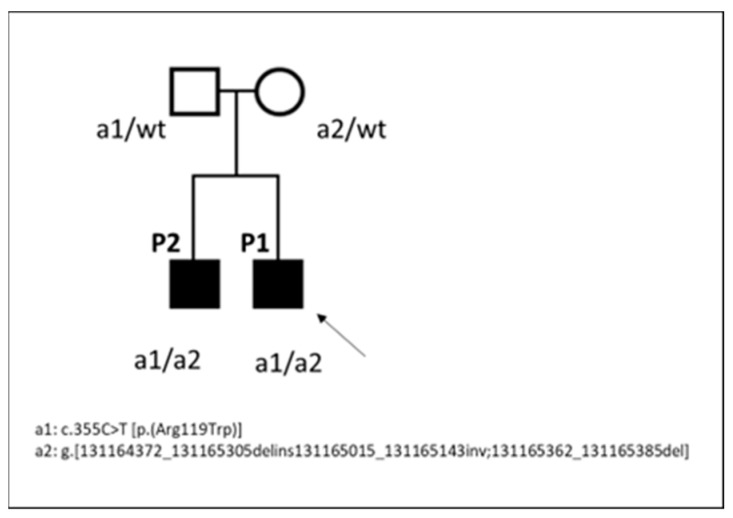
Pedigree of family; affected family members are shown in black. Circles indicate females and squares males. The Proband P1 is indicated by the arrow. *HINT1* genotypes are indicated below the symbols (a1, missense variant; a2, complex rearrangements; wt, wildtype). The patients are compound heterozygous (a1/a2), whereas the father is heterozygous for the a1 variant, and the mother is heterozygous for the a2 variant.

**Figure 5:**
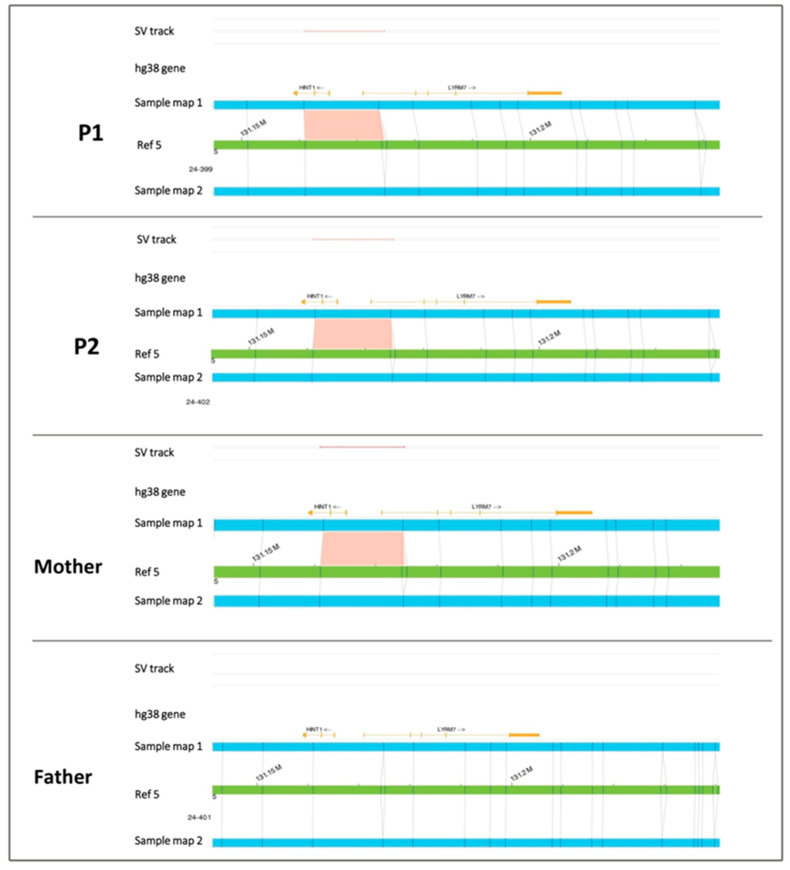
Identification and visualization of the deletion by OGM. Reference map: green bar; sample map: blue bar. OGM revealed a heterozygous ~800 bp deletion (pink bar in the SV track) in the patient [ogm[GRCh38] 5q23.3(131160930_131174751)x1], his brother [ogm[GRCh38] 5q23.3(131160943_131174737)x1], and mother [ogm[GRCh38] 5q23.3(131160926_131174755)x1] compared to the reference genome (Ref hg38, green bar). The deletion, absent in the father, was located within a range of 13 Kb (chr5:131,160,929–131,174,750 hg 38), partially involving *HINT1* and *LYRM7.*

NGS analysis in P1 revealed (Figure 3A) a heterozygous missense variant c.355C>T [p.(Arg119Trp)] in *HINT1* (NM_005340.7), and no other variants (pathogenic or VUS) were present in this gene. According to the ACMG guidelines, the c.355C>T variant can be classified as likely pathogenic (evidence of pathogenicity: PM1, PM2, PP2, PP3, PP5). Sanger sequencing (Figure 3B) in the family corroborated the presence of this variant in P1 and his affected brother and showed its paternal inheritance (Figure 4). The variant c.355C>T (p.Arg355Trp) results in damage in several prediction sites (SIFT, Polyphen 2, AlphaMissense, REVEL, ClinPred, Meta SVM, Meta LR) and is affecting a highly conserved residue. CADD score is 25. It has also been previously reported in a patient with neuromyotonia and axonal neuropathy [16]. A visual inspection of the *HINT1* region in P1 using IGV highlighted a possible intragenic complex rearrangement, including the 5′ UTR, exon 1, and intron 1. Further examination of the family using OGM highlighted the presence of a heterozygous deletion at 5q23.3 involving *HINT1*; the deletion was ~800 bp in size in both children and their mother (Figure 5). Finally, Sanger sequencing confirmed the rearrangement’s complexity as g.[131164372_131165305delins131165015_131165143inv;131165362_131165385del] (NC_000005.10) (Figure 3C).

This novel variant most likely results in a functional loss of one gene copy, but unfortunately, we had no residual blood or muscle tissue available to test effects at the cDNA level. The complex rearrangement involves the entire coding sequence of exon 1, including part of the 5′ UTR and the ATG initiation site, and is probably pathogenic for the following reasons: (1) it is not reported in the DGV database (https://dgv.tcag.ca/, accessed on 31 January 2024); (2) it involves exon 1 containing the initiation codon; therefore, it is expected to abolish the function of HINT1 by using an alternative upstream or downstream initiation site, thus modifying the amino acid sequence of the protein; (3) segregation analysis shows this variant in the affected sibling.

## 4. Discussion

*HINT1*, located on chromosome 5q23.3, codes for a homodimeric purine phosphoramidase ubiquitously expressed but particularly both in central (CNS) and peripheral nervous system (PNS) tissues. *HINT1* protein is involved in the function of the nervous system. In neurons, it stabilizes the interaction of different receptors and regulates the effects of their disturbances in axonal signaling. Furthermore, *HINT1* is also known to regulate transcription factors involved in tumor progression and apoptosis [17].

Loss of function mutations in *HINT1* are associated in humans with autosomal recessive axonal neuropathy with neuromyotonia (ARAN-NM) [8], probably due to the involvement of the gene product in neuronal signaling pathways [18]. To date, 28 pathogenic variants have been described (https://franklin.genoox.com/clinical-db/home (accessed on 31 January 2024)), 14 nonsense and 14 missense/inframe indel. Our patients harbor heterozygous compound variants in *HINT1*: a c.355C>T [p.(Arg119Trp)], paternally inherited and already described in the literature as a pathogenic [16] and a novel, maternally inherited complex rearrangement located in the region 5′UTR-exon 1-intron 1, possibly resulting in functional loss of one gene copy.

The complex rearrangement of the maternal chromosome found in our subjects was initially overlooked by routine NGS data analyses. However, clinical evidence suggested a phenotype compatible with ARAN-NM, requiring further analyses. Upon revisiting the case and combining all reports of tests in this family, both clinicians and geneticists agreed to further explore the *HINT1* region. This allowed the identification of the second causative variant in the family.

Clinically, our patients share characteristics already reported in the literature [19] in support of the pathogenicity of the mutation. Similarly to previous descriptions [20], both patients presented symptoms onset during late childhood/early adolescence, with progressive weakness, motor impairment, beginning of retractions, and foot deformities. Both of our subjects exhibited neurodevelopmental symptoms consistent with the assumption of CNS involvement [21]. The association between *HINT1* mutations and neuropsychiatric disorders is not fully elucidated. It has already been demonstrated that an alteration of mRNA/*HINT1* expression occurs in post-mortem brains of patients with mood disorders, specifically in those with a diagnosis of bipolar disorder [22] and major depressive disorder [23]. These findings suggest a possible role of *HINT1* in emotional regulation. Manic-like behaviors were also observed in *HINT1*-KO mice [24]. Moreover, a possible gender-specific (male) association between *HINT1* and schizophrenia has been hypothesized [25]. Possible influences on anxiety levels are still under investigation since the pertinent literature shows evidence for reduced [24] or elevated [7] levels of anxiety in *HINT1*-KO mice.

Only in recent years have neuropsychiatric manifestations in ARAN-NM patients been reported, such as cognitive and language impairment, learning difficulties, behavioral issues, mood and anxiety disorders, ADHD, and OCD [3,26,27,28].

Serum CK levels were mild-to-moderately elevated in the two brothers we reported, probably due to persistent neurogenic muscle atrophy. The nerve conduction studies showed both features consistent with axonal neuropathy, once again in agreement with the literature data [29]. No EMG examination has yet been performed, but the neuromyotonic phenomenon was clinically evident in both children, and its detection was crucial to direct the diagnostic approach.

In HINT1-related disorder, muscle biopsy usually shows neurogenic features [30], but the presence of rimmed vacuoles without inflammation has also been described [31] as a possible consequence of active denervation or a potential alteration of a myopathic evolution. At the present time, we have planned skin and skeletal muscle biopsies in both patients. To summarize, this study reiterates the importance of a multidisciplinary approach in the field of neurogenetic disorders and suggests the necessity for more sensitive techniques for the evaluation of first-instance unclear cases.

## 5. Conclusions

NGS analysis can provide specific genetic diagnoses for a subset of patients with CMT/HN disorders, which improves disease and genetic counseling and prepares patients for disease-focused therapies. Despite these advancements, many patients with known or suspected CMT/HN disorders remain without a specific genetic diagnosis. Continued advancements in genetic testing and a better understanding of genotype/phenotype correlation will further improve detection rates for patients with suspected CMT/HN disorders; however, as this case highlights, clinical assessment must continue to be of primary importance in guiding and interpreting genetic analyses. The complex rearrangement in the two siblings could have been overlooked if the phenotype had not been evaluated as a whole (from a clinical, neurophysiological, and neuroimaging point of view). However, more studies are necessary to elucidate the effect of this variant on gene products and its exact consequences.

## Figures and Tables

**Table 1:** Cognitive profile and behavioral assessments.

	P1	P2
**Cognitive assessment (WISC-IV)**		
IQ	45	65
VCI	62	80
PRI	58	74
WMI	64	76
PSI	50	62
**CBCL 6–18**		
Syndrome scales	Anxious/Depressed, nc	Anxious/Depressed, nc
	Withdrawn/Depressed, C (T = 76)	Withdrawn/Depressed, nc
	Somatic Complaints, nc	Somatic Complaints, nc
	Social Problems, B (T = 67)	Social Problems, nc
	Thought Problems, C (T = 73)	Thought Problems, nc
	Attention Problems, nc	Attention Problems, nc
	Rule-Breaking Behavior, C (T = 70)	Rule-Breaking Behavior, nc
	Aggressive Behavior, C (T = 72)	Aggressive Behavior, nc
DSM-Oriented scales	Affective Problems, C (T = 75)	Affective Problems, nc
	Anxiety Problems, nc	Anxiety Problems, nc
	Somatic Problems, nc	Somatic Problems, nc
	ADHD Problems, B (T = 66)	ADHD Problems, nc
	Oppositional Defiant Problems, B (T = 66)	Oppositional Defiant Problems, nc
	Conduct Problems, C (T = 70)	Conduct Problems, nc
**MASC 2**		
Parent report	Separation anxiety, nc	Separation anxiety, nc
	Generalized anxiety, B (T = 65)	Generalized anxiety, B (T = 65)
	Social anxiety, B (T = 69)	Social anxiety, nc
	Humiliation/rejection, nc	Humiliation/rejection, B (T = 65)
	Prestational fear, C (T = 70)	Prestational fear, nc
	Obsessive-compulsive, nc	Obsessive-compulsive, C (T = 78)
	Physical symptoms, nc	Physical symptoms, C (T = 72)
	Panic, nc	Panic, B (T = 68)
	Tension/agitation, B (T = 68)	Tension/agitation, C (T = 72)
	Avoidance of danger, nc	Avoidance of danger, nc
	Total, B (T = 66)	Total, C (T = 74)

[B: borderline, C: clinical, nc: non-clinical; WISC-IV: Wechsler Intelligence Scale for Children-IV; VCI: verbal comprehension index; PRI: perceptual reasoning index; WMI: working memory index; PSI: processing speed index; CBCL 6–18: Child Behavior Checklist 6–18; MASC 2: Multidimensional Anxiety Scale for Children].

**Table 2:** Nerve conduction study showing in P1 a reduction in motor response amplitude of bilateral extensor digitorum brevis (EBD) and in P2 a reduction in motor response amplitude of left EBD and abductor allucis and a reduction in sensitive response amplitude of left suralis.

	P1			P2		
**Motor conduction Velocity**	**Latency (ms)**	**Amplitude (mV)**	**MCV (m/s)**	**Latency (ms)**	**Amplitude (mV)**	**MCV (m/s)**
**Right Medianus**						
Wrist-Thumb	-	-	-	3.2	9	
Elbow-Thumb	-	-	-	8.7	9	51.8
**Right Ulnaris**						
Wrist-ABM	2.7	15		2.8	16	
Elbow-ABM	6.8	15	56.1	8.0	16	51.9
**Right Tibialis**						
Medial malleolus-Abductor allucis	3.2	5		-	-	-
Popliteal fossa-Abductor allucis	12.3	5	40.7	-	-	-
**Left Tibialis**						
Medial malleolus- Abductor allucis	-	-	-	4.0	0.3	
Popliteal fossa- Abductor allucis	-	-	-	13.8	0.3	43.9
**Right Peroneus profundus**						
Foot dorsum-EBD	4.2	0.7		-	-	-
Caput-EBD	12.3	0.7	42.0	-	-	-
**Left Peroneus profundus**						
Foot dorsum-EBD	4.2	0.3		5.5	0.5	
Caput-EBD	12.2	0.3	40.0	15.0	0.5	40.5
**Sensitive Conduction Velocity**	**Latency (ms)**	**Amplitude (μV)**	**SCV (m/s)**	**Latency (ms)**	**Amplitude (μV)**	**SCV (m/s)**
**Right Medianus**						
Palm-III finger	-	-	-	1.3	53	56.0
Wrist-Palm	-	-	-	2.3	47	57.1
Elbow-Wrist	-	-	-	6.9	18	62.0
**Right Ulnaris**						
Wrist-V finger	1.6	76	54.5	1.9	43	52.1
Elbow-Wrist	5.7	30	56.8	6.7	18	56.5
**Right Suralis**						
Third medial Gastrocnemius- Lateral malleolus	2.1	29	42.9	-	-	-
**Left Suralis**						
Third medial Gastrocnemius- Lateral malleolus	-	-	-	3.0	7	40.0

## Data Availability

The original contributions presented in this study are included in the article. Further inquiries can be directed to the corresponding author.
